# *Plectus* - a stepping stone in embryonic cell lineage evolution of nematodes

**DOI:** 10.1186/2041-9139-3-13

**Published:** 2012-07-02

**Authors:** Jens Schulze, Wouter Houthoofd, Jana Uenk, Sandra Vangestel, Einhard Schierenberg

**Affiliations:** 1 Biocenter, University of Cologne, Zülpicher Strasse 47b, Cologne, 50674, Germany; 2 Department of Biology, Ghent University, Ledeganckstraat 35, Ghent, 9000, Belgium

**Keywords:** Nematode, embryogenesis, cell lineage, cell specification, evolution, developmental system drift, *Plectus*, *C. elegans*

## Abstract

**Background:**

Recent studies have challenged the widespread view that the pattern of embryogenesis found in *Caenorhabditis elegans* (clade 9) is characteristic of nematodes in general. To understand this still largely unexplored landscape of developmental events, we set out to examine more distantly related nematodes in detail for temporospatial differences in pattern formation and cell specification. Members of the genus *Plectus* (clade 6) seem to be suitable candidates to show variety, with certain idiosyncratic features during early development and the convenient availability of cultivatable species.

**Methods:**

The study was conducted using 4-D lineage analysis, 3-D modeling of developing embryos and laser-induced ablation of individual blastomeres.

**Results:**

Detailed cell lineage studies of several *Plectus* species reveal that pattern formation and cell fate assignment differ markedly from *C. elegans*. Descendants of the first somatic founder cell S1 (AB) - but not the progeny of other founder cells - demonstrate extremely variable spatial arrangements illustrating that here distinct early cell-cell interactions between invariant partners, as found in *C. elegans*, cannot take place. Different from *C. elegans*, in *Plectus* alternative positional variations among early S1 blastomeres resulting in a ‘situs inversus’ pattern, nevertheless give rise to adults with normal left-right asymmetries. In addition, laser ablations of early blastomeres uncover inductions between variable cell partners.

**Conclusions:**

Our results suggest that embryonic cell specification in *Plectus* is not correlated with cell lineage but with position. With this peculiarity, *Plectus* appears to occupy an intermediate position between basal nematodes displaying a variable early development and the *C. elegans*-like invariant pattern. We suggest that indeterminate pattern formation associated with late, position-dependent fate assignment represents a plesiomorphic character among nematodes predominant in certain basal clades but lost in derived clades. Thus, the behavior of S1 cells in *Plectus* can be considered an evolutionary relict in a transition phase between two different developmental strategies.

## Background

The phylum Nematoda is considered a very ancient phylum reaching back into the Precambrian [1-3]. Well-preserved fossils of nematodes from the early Devonian very much resemble recent basal representatives [4]. The number of extant nematode species is large with estimates ranging from tens of thousands to several millions [5-8]. Their adaptation to nearly all environmental conditions would predict a high plasticity on the morphological and physiological level. But the nematode body plan has remained uniform, despite a high genetic divergence and evolutionary variations in core developmental pathways even between close relatives [9-11].

With respect to embryonic development, the strong similarities in cleavage pattern between the classic study object *Ascaris megalocephala* (today: *Parascaris equorum*; [12,13]) and the modern model system *Caenorhabditis elegans*[14] led to the notion that this represents how nematodes generally develop. Extended studies in representatives from different branches of the phylogenetic tree made clear, however, that despite strong conservation of morphology, their embryogenesis is surprisingly variable [7,15-17] and hence the *Ascaris*/*C. elegans* pattern constitutes only one way to generate a worm.

Differences in early development among nematode species invalidate the long-standing notion formulated by von Baer [18] that during ontogeny first general characters are expressed that need to be conserved before evolutionary novelties are added.

Our recent analysis of basal nematodes (clades 1 and 2, according to the phylogenetic classification by [19] subdividing nematodes into 12 clades) revealed considerable differences from *C. elegans*, including lack of early asymmetric divisions associated with soma/germline separation, but also establishment of bilateral symmetry, spatial cell arrangements [17]. Thus, nematodes appear to be an attractive taxon to study evolution of development.

Representatives particularly amenable for comparative embryonic studies should meet several criteria: simple culture conditions, fast development and transparent eggs. So far, only free-living members of clades 6 to 12 seem to fulfill these criteria [17]. In search of candidates from these clades that may show developmental characteristics of both, basal nematodes (see above) and more derived species (clades 7 to 12; resembling the pattern found in *Ascaris* and *C. elegans*) representatives of the order Plectida (clade 6; [7]) appear to be promising candidates to search for such a combination of plesiomorphic and apomorphic characters. More than 70 different plectid species have been described [20] showing characteristic morphological features like setae (bristles) distributed over the entire body and unispiral amphids (chemosensory organs). The phylogenetic classification of plectid nematodes varied considerably in the past [21]. Several members of the genus *Plectus* can be cultured in the laboratory and embryos are transparent enough with sufficiently rapid development to allow detailed lineage analysis [22]. Among these, *Plectus sambesii* was found to have the fastest life cycle and highest reproductive rate under standard growth conditions. Therefore, most studies described here were performed on this representative, but other members of the genus were analyzed as well.

In the *C. elegans* embryo, cell specification requires reproducible spatial positioning of cells at specific milestone times to allow contacts and inductive signaling between neighboring blastomeres [23-25]. On the one hand, several plectid species were found to follow an early cleavage pattern similar to *C. elegans,* including the series of asymmetric cleavages going along with soma/germline separation. On other hand, plectids are clearly different in displaying an essentially perfect early bilateral symmetry within individual lineages and by initiating gastrulation prematurely with the immigration of an undivided gut founder cell [22].

In *C. elegans*, an invariant early left/right asymmetry is established in the 6-cell stage when two S1(AB) cell cousins on the left move anteriorly, while the corresponding pair on the right side moves posteriorly [26]. As a result, cells occupying equivalent positions in the left and right branches of the lineage tree adopt nonequivalent fates, and, vice versa, equivalent cell fates in the left and right half of the embryo are executed in a complex manner by cells of nonequivalent position in the lineage [14]. In the hatched worm, these early events result in a dextral handedness of organs. By experimentally inverting the left/right asymmetry of the four AB cells, a perfectly healthy ‘situs inversus’ (inverted left/right asymmetries) can be generated [27,28]. As the left/right shift of early blastomeres, which in *C. elegans* is a necessary prerequisite for the typical pattern of inductive interactions, was not observed in *Plectus*[22], we wondered whether cell specification follows different rules in this genus. Therefore, we set out to compare cell lineages, spatial pattern formation and cell fate assignment in *Plectus* with those of *C. elegans* and representatives of basal clades in order to better understand the evolution of developmental processes during embryogenesis of nematodes.

## Methods

### Strains and culture

The strains *Acrobeloides maximus* (DF5048), *Acrobeloides nanus* (ES501), *Aphelenchus avenae* (RGD103)*, Bursaphelenchus xylophilus* (S10), *Caenorhabditis brenneri* (SB280), *Caenorhabditis briggsae* (AF16), *Caenorhabditis elegans* (N2), *Choriorhabditis dudichi* (SB122), *Diploscapter* sp. (JU359), *Diploscapter coronatus* (PDL0010), *Halicephalobus gingivalis* (JB128), *Panagrolaimus* sp. (JU765), *Panagrolaimus superbus* (DF5050), *Plectus aquatilis* (PDL0018), *Plectus minimus* (PDL0012), *Plectus sambesii* (ES601), *Plectus* sp. (ES603), *Pristionchus pacificus* (PS312), *Protorhabditis* sp. (DF5055), *Protorhabditis* sp. (JB122), *Rhabditis belari* (ES103), *Rhabditis dolichura* (ES101), *Teratocephalus lirellus* (JB049), *Tylocephalus* sp. (PDL1001) and *Zeldia punctata* (PDL0003) were cultured at 23°C on minimal agar plates essentially as described in [22]. Strains not isolated in our laboratory (ES) were obtained from Paul de Ley and Jim Baldwin, University of California at Riverside, USA; Marie-Anne Felix, Université Jacques Monod, Paris, France; Walter Sudhaus, Freie Universität Berlin, Germany; and Ralf Sommer, MPI for Developmental Biology, Tübingen, Germany. Data for *Ascaris* were taken from [12,13], for *P. marina* from [29], and for *H. gingivalis* from [30]. Recordings of *Meloidogyne incognita* were obtained from Bartel Vanholme and Alejandro Calderón-Urrea, California State University, Fresno, USA.

### Cell nomenclature and cell fate assignments

Projection of *C. elegans* standard cell nomenclature [14,31] onto other nematode species would imply similar cell fate patterns. Therefore, we apply neutral lineage names (S1to S4, somatic founder cells; P1 to P4, germline). In *C. elegans*, but not necessarily in other nematodes S1 = AB, S2 = EMS, S3 = C, S4 = D [32]. For easier comparison between *Plectus sambesii* and *C. elegans*, we sometimes added for the former the standard names in parentheses.

Although we did not trace all embryonic divisions, we followed the behavior and localization of cells far enough to determine the tissues to which they contribute [17], in particular with respect to pharynx and hypodermis formation.

### 4-D microscopy

Early stage embryos collected from culture plates or cut out of gravid adults were mounted on slides carrying a thin 3% agarose layer as a mechanical cushion [17]. Alternatively, gravid nematodes were dissected with a scalpel in a drop of distilled water on a poly-lysine-coated coverslip (0.01%) with a drop of petroleum jelly as spacer in all four corners (modified after [33]). The coverslip was sealed with melted petroleum jelly. Embryonic development was examined with DIC optics using a 100x objective. Stacks of optical sections were digitally recorded at 30 to 60 second intervals and at 23°C with a 4-D microscope. 3-D tracing of cell behavior and generation of cell lineages were software-supported (Simi Biocell, Unterschleissheim, Germany), originally described in [34]. If not stated otherwise, development of at least seven embryos per species were analyzed.

### Cell ablation experiments

1-cell embryos of *P. sambesii* were prepared for 4-D microscopy as mentioned above. For cell ablation, a Spectra-Physics Explorer™ Q-switched solid-state laser (Newport; Darmstadt, Germany) coupled to a Leica DMLB microscope (Leica; Wetzlar, Germany) via reflector optics was used. P1 and P2 cells were irradiated 3 × 45 seconds (at 50 Hz, 5 μJ) with 30-second intervals between irradiation periods. S3 cells were irradiated 3 x 30 seconds (at 50 Hz, 2 μJ) with 30-second intervals.

### Cell extrusion experiments and cell culture

Manipulated embryos were cultured in embryonic growth medium (EGM) adopted from [35] to support closure of the vitelline membrane [36] necessary for normal development. Perforated embryos continued to develop to several hundred cells. In contrast to *C. elegans*, in *P. sambesii* this medium does not support differentiation of isolated blastomeres outside the protective vitelline membrane. 2-cell embryos of *P. sambesii* were mounted on poly-lysine-coated slides, and distilled water was then replaced by EGM. A hole was burned into the posterior part of the eggshell by pulsing repeatedly with the Spectra-Physics Explorer™ Q-switched solid-state laser (1 Hz, 60 μJ). By using gentle pressure with a needle on the coverslip, the P2 cell was squeezed out of the eggshell and detached from the remainder of the embryo [37]. To visualize endocytotic activity as a marker for gut differentiation, EGM was supplemented with 0.25% Lucifer Yellow VS (LY, Mr 550, Sigma-Aldrich; Steinheim, Germany) and was allowed to penetrate the embryo by puncturing the eggshell with a laser microbeam [38]. After 15 minutes’ incubation time, the LY medium was replaced by regular EGM. Localization of LY was visualized in terminal phenotypes by confocal microscopy.

## Results

### Cleavage pattern in the P1 lineage is conserved between *P. sambesii* and *C. elegans*

**Figure 1:**
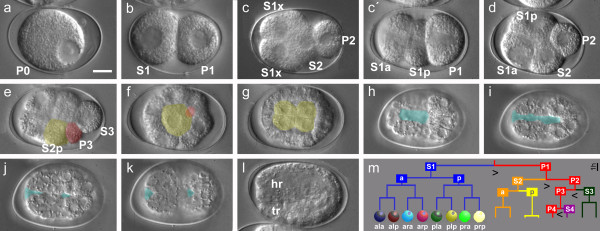
**Embryogenesis in *Plectus sambesii* **. **(a)** 1-cell stage (P0) with single pronucleus; **(b)** 2-cell stage with larger somatic founder cell S1 and smaller germline cell P1; **(c)** T-shaped 4-cell stage after transverse division of S1 and longitudinal division of P1; **(c’)** rarely S1 divides with longitudinal spindle orientation; **(d)** diamond-shaped 4-cell stage after rearrangement of blastomeres; **(e)** 8-cell stage, with gut precursor S2p (yellow) and germline cell P3 (red); **(f)** approximately 16-cell stage, S2p (yellow) has migrated to the center but keeps contact with the primordial germ cell P4 on the surface (red; mostly out of focus); **(g)** embryo with 4-cell gut primordium (yellow); **(h-k)** formation and closure of blastopore (blue) on the ventral surface of the developing embryo; **(l)** twofold stage (early morphogenesis) with future head region (hr) top and tail region (tr) bottom; **(m)** early cell lineage, divisions of germline (P1 to P4; changing position indicates ‘polarity reversal’ as described for *C. elegans*; Schierenberg, 1987) generate somatic founder cells (S1 to S4), the eight descendants of S1 are color coded; S2 divides into pharynx/muscle precursor S2a (orange) and gut precursor S2p (yellow). Arrowheads indicate asymmetric divisions. DIC optics. Orientation: anterior, left. Bar, 10 μm.

**Figure 2:**
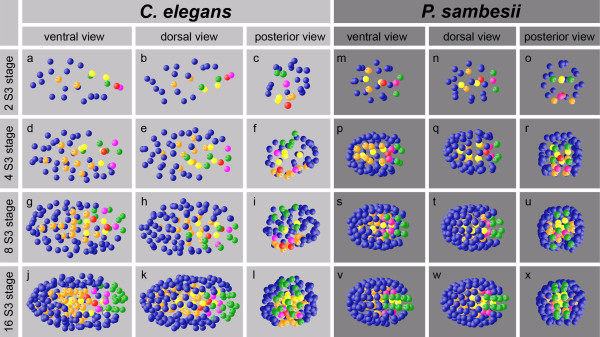
**Formation of bilateral symmetry.** Three-dimensional models of embryonic cell nuclei. In *C. elegans*, symmetry formation within individual lineages is not very apparent in early stages due to oblique division of somatic founder cells, but becomes successively more obvious even though not perfect. This is exemplified by the behavior of the S3 cells (green spheres; **(a-l)**). In contrast, *P. sambesii* performs strict left-right divisions of founder cells, generating in this way an essentially perfect bilaterally symmetric early embryo **(m-x)**. Color code: S1 (AB), blue; S2a (MS), orange; S2p (E), yellow; S3 (C), green; S4 (D), purple, germline (P), red.

In *P. sambesii*, consecutive asymmetric cleavages of the germline (P0 to P3) along the main body axis result in the germ cell precursor P4 and somatic founder cells S1 to S4; (Figure1; for cell nomenclature see Methods). Transverse orientation of the S1 and longitudinal orientation of the P1 cleavage spindles are like in *C. elegans*. Thus, both species follow the T1-type of cleavage (Figure1c; [17]). In rare cases (<1%), longitudinal orientation of the cleavage spindle in S1 (n >200; Figure1c’) results in the I2-type. Both variants, however, result in a rhomboid 4-cell stage (Figure1d) as found in *C. elegans* and many other nematodes [17] and lead to hatching juveniles. Our observations are in line with an earlier description of embryogenesis in *P. sambesii*[22] with respect to cell behavior of P1 descendants. Characteristic differences to *C. elegans* (Figure2a-c) are found in the formation of bilateral symmetry in the S2a (MS) and S3 (C) lineages, where the first division generates two daughters in strictly left and right positions (Figure2m-o) and gastrulation is initiated earlier with the immigration of the single gut founder S2p (E; Figure1f) rather than its daughters.

We conclude from our study of a large number of *P. sambesii* embryos (n = 73) that behavior of the P1 descendants is essentially invariant and similar to that of *C. elegans*[14,33,34].

### Endoderm specification requires induction by P2

In *C. elegans*, gut (E) differentiation depends on an inductive signal between P2 (germline) and S2 (EMS) in a narrow time window in the early 4-cell embryo [39,40]. Because of the differences in gastrulation (see introduction) we wondered whether in *P. sambesii* gut-specific differentiation depends on an induction by the germline, as found in *C. elegans*.

**Figure 3:**
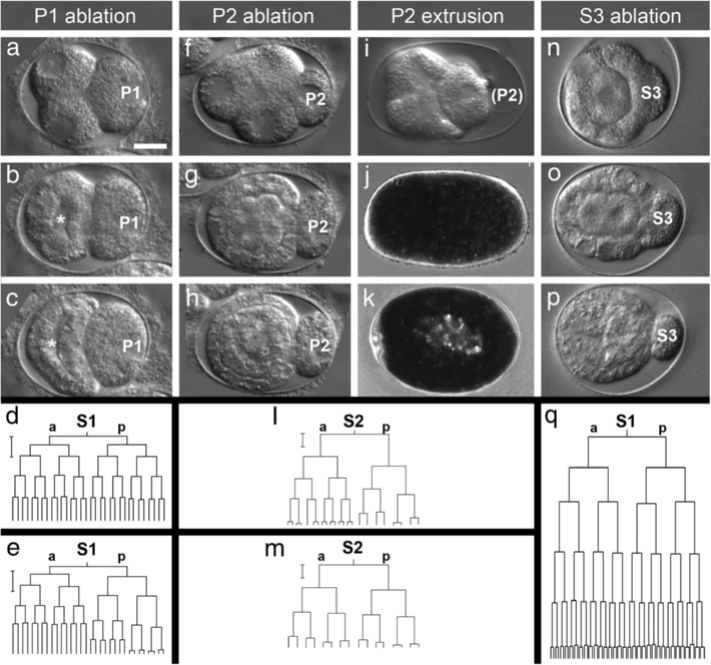
**Cleavage behavior after cell elimination in *P. sambesii* . (a-c)** After ablation of P1, the S1 descendants divide and form a blastocoel (marked with asterisk); **(d, e)** after ablation of P1, the S1 descendants can express normal synchronous cell cycles (d) or S1p descendants cleave asynchronously (e); **(f-m)** after early P2 ablation, S2 descendants lose their ability for asynchronous cell cycles (m) but after late ablation the normal cell cycle differences are expressed (l); (i-k) not after early (j) but after late extrusion of P2 (i), fluorescent marker dye accumulates in the gut primordium (k); **(n-q)** after early ablation of S3 (or P1/P2, see text), the typical retardation of cell cycles in three S1 descendants (see Figure 6) is lost (q). a-i, n-p: DIC optics; j, k, epifluorescence. Bar, 10 μm.

We therefore ablated the P2 cell (n = 10; Figure3f) as soon as it had separated from its sister cell S2 (EMS). In 9/10 cases we observed a typical early gastrulation with immigration of a single gut precursor (Figure3g,h) and separation into a faster dividing S2a (MS) and a slower S2p (E; Figure3l). In a single case (1/10) we found a more or less synchronous sequence of divisions (Figure3m). Visualization of gut differentiation was difficult as live markers like autofluorescence or formation of birefringent gut granules [41-43] are weak or absent.

Since in *C. elegans*, physical separation of P2 in time from the remainder of the embryo reliably prevents gut induction [39,40], we removed the germline blastomere in the early 4-cell stage through a laser-induced hole in the eggshell (n = 3; Figure3i). In these experiments, we added Lucifer Yellow to the extrusion medium, as this fluorescent marker dye is quickly and specifically taken up by gut precursor cells [38,41]. In test experiments, we had determined that such an accumulation takes place in *Plectus* as well. After early P2 extrusion (n = 3), division of S2a and S2p was found to be essentially synchronous (Figure3m) in contrast to normal development. In addition, although the typical early immigration of the gut precursor cell took place, there was no accumulation of fluorescent dye in the descendants of this cell (Figure3j), indicating the absence of typical gut differentiation. To determine the time window of the assumed signaling, we then repeated this experiment in the late 6-cell stage after the division of the two S1 (AB) daughters (n = 2). Under these conditions, cell cycle lengths of S2a (MS) and S2p (E) became different from each other (Figure3l), normal internalization of S2p (E) took place, and fluorescent dye accumulated in the gut primordium (Figure3k).

Thus, our data show that, like in *C. elegans*, in *P. sambesii:* (i) P2 is necessary during the early phase of the S2p (E) cell cycle for proper endoderm specification and (ii) ablation of P2 is not sufficient to inhibit reliably its inductive signaling.

### High positional variation of S1 cells in *P. sambesii* unlike in *C. elegans*

During embryonic development of *C. elegans*, but also *P. sambesii* (Figure1; [22]) pattern formation among P1 descendants takes place in a simple and invariant fashion. In contrast, in *C. elegans*, spatial arrangement of blastomeres and fate designation in the S1 (AB) lineage, even though invariant, is rather complex [14] and requires specific cell-cell interactions within narrow time windows [25,39]. Based on our observation that pattern formation in the S1 lineage of *Plectus* differs from *C. elegans*[22], we started a more detailed 4-D analysis of embryonic S1 lineages in *P. sambesii*, including an evaluation of cell-cell contacts, spatial variations thereof and their effect on cell fate assignment (n = 73).

Hench *et al*. [33] reported recently that squeezing *C. elegans* embryos between microscope slide and coverslip can influence the spatial arrangement of blastomeres without necessarily compromising normal development. Therefore, we applied their minimal pressure-mounting technique for 30/73 embryos and for the remaining 43/73 embryos our standard procedure with an agarose pad as a cushion (see Methods). We found that the ratios of different spatial variants (described below) were not affected by the mounting technique applied.

**Figure 4:**
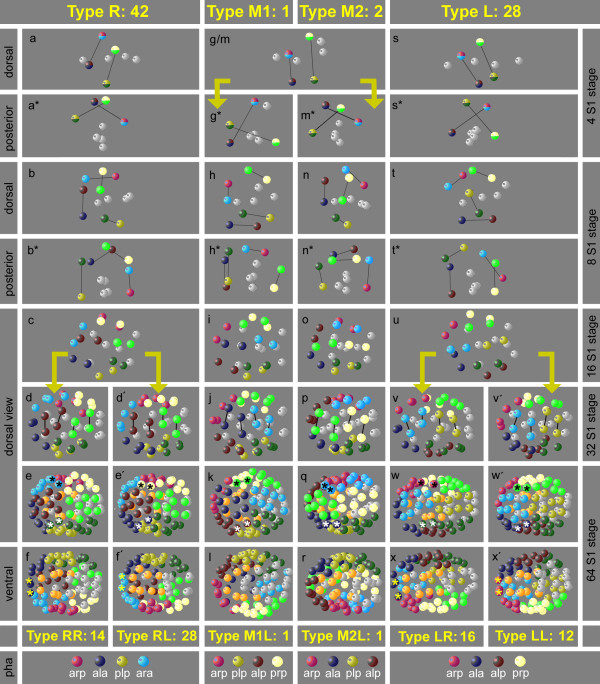
**Variable arrangement of S1 descendants.** Three-dimensional models of embryonic cell nuclei. With respect to the spatial arrangement of S1 blastomeres, four different cleavage types can be defined in the 8-cell stage (R, S1 blastomeres on the *right* side are positioned posterior to their left counterparts **(a)**; L, S1 blastomeres on the *left* side are positioned posterior to their right counterparts **(s)**; M1/M2, with respect to anterior/posterior S1 blastomeres on both sides occupy equivalent positions **(g/m)**, but with respect to dorsal/ventral they occupy opposite positions (g*, m*). With the division of 16 S1 cells, cleavage types R and L each reproducibly split into two further spatial variants **(d + d’**; **v + v’)** due to shifts of left and right S1 descendants relative to each other. To better visualize that in the 64 S1 cell stage, specific positions on the left and right sides **(e-w’)** and at the anterior pole **(f-x’)** of the embryo are occupied by blastomeres from variable lineage origin, selected cells are marked with asterisks. Color code: members of the eight S1 descendants are marked as shown in Figure 1. Non-S1 cells are shown in light grey.

We obtained the following results. First, minor deviations in cell cycle lengths and the sequence of cell divisions are within the same range as found in *C. elegans*[34] and therefore are not considered further here. Second, the descendants of the eight S1 sublineages (ala, alp, ara, arp, pla, plp, pra and prp; Figure1m) form and preserve coherent well-defined regions as found in *C. elegans*[33,34,44,45]. Third, in contrast to the invariant embryonic development of *C. elegans* (called ‘monomorphic’ in the following), the spatial arrangement of these eight regions in *P. sambesii* is considerably variable (called ‘polymorphic’ in the following). The different regions do not intermingle, variants do not merge during ongoing development, and all of these are compatible with development to a fertile normal worm. They can be classified into distinct patterns (‘polymorphs’) as shown in Figure4. Already in the 4 S1-cell stage, we can define four different variants by position of the S1 descendants (Types R, M1, M2 and L; Figure4a, g, m, s). In both type M variants, S1a and S1p (Figure4g, m) divide with transverse spindle orientations, generating left and right descendants that keep their transverse positions. These types are rare (n = 3/73) and early differences between M1 and M2 polymorphs become obvious only in a posterior view (Figure4g*, m*). Type R (Figure4a, a*) reveals a prominent shift of the two right S1 descendants to the posterior, while their siblings on the left side occupy more anterior positions. This type is the most abundant (n = 42/73). Members of type L (Figure4s, s*) behave the other way round such that left S1 descendants become located posterior relative to the right S1 cells. This type was found frequently (n = 28/73).

During further development, three of the four above described polymorphs (Figure4a, m, s) were found to split into two further variants due to minor differences in orientation of certain cleavage spindles in the 16 S1-cell stage (Figure4d + d’, v + v’; corresponding variant of Figure4p not shown). Nevertheless, the general positions of the eight S1 cell clones relative to each other do not vary. While type R (a) and L (s) are frequent, the two variants of type M (g*, m*) are rare. In type L, the variants LR (n = 16) and LL (n = 12) occurred at about the same frequency, polymorph RL (n = 28) was twice as frequent as variant RR (n = 14). The rare types M2L (n = 1; Figure4p-r) and M2R (n = 1; not shown) occurred with the same low frequency. A corresponding equivalent to the single M1L variant (Figure4j-l) was not found in our collection.

### Are polymorphic patterns genetically inherited?

As our studied *P. sambesii* strain reproduces parthenogenetically [46] and therefore genetic traits do not mix, we wondered whether the population is genetically diverse such that individuals with distinct developmental programs leading to one specific spatial pattern in their embryos coexist in fixed ratios.

To look for this, we recovered embryos whose early embryogenesis had been analyzed and allowed them to hatch and produce eggs. Samples consisting of multiple second-generation embryos from these single identified isolated mothers were noted and the eggs derived from each individual worm were studied to determine whether such eggs showed only the developmental pattern of their mother, or if one mother could produce different polymorphs. Among eggs from a mother of type M2R, we found 2 x LR, 2 x LL, 1 x RR, 4 x RL; in eggs from a mother of type LR we found 1 x LL, 2 x RR, 5 x RL; and in eggs from a mother of type RR, we found 4 x LR, 1 x LL, 1 x RR. These data demonstrate that in *P. sambesii* the different embryonic patterns are not inherited as exclusive genetic traits. Despite the small sample size, it appears likely that a mother can produce any and all of the observed polymorphs.

### Differential behavior of S1 descendants depends on the P1 lineage

Our findings demonstrate that in the polymorphs of *P. sambesii*, cell positions and therefore cell-cell contacts of S1 descendants vary. This raises the question whether cell fate assignment in the S1 lineage can take place in the same way as in the monomorphic *C. elegans* in which a strict correlation with lineage origin exists [14] and specific inductions between individual cells are essential [23-25].

As earlier studies on another nematode, *Acrobeloides nanus* (clade 11), had shown an altered cleavage and differentiation pattern of S1 descendants after elimination of P1 [47,48], we performed the same test with *P. sambesii*. After ablation of P1 (n = 11; Figure3a), the S1 descendants divide in a symmetric fashion and form a prominent blastocoel (Figure3b, c). No events resembling gastrulation (as we had found in *A. nanus*[47,48] and *R. culicivorax*[32]) were observed and the terminal phenotype did not show muscle contractions as found in experimental *A. nanus* embryos. However, with respect to cell cycle durations, manipulated *Plectus* embryos can be subdivided into two groups. In the first one (n = 5), descendants of S1 divided more or less synchronously (Figure3d) but, in contrast to the untreated embryo, the typical later retardation in parts of the S1 lineage was not found (see hypodermis specification below). In the second group (n = 6), early descendants of S1p behaved atypically in that they expressed retarded and differential cell cycles (Figure3e) while S1a descendants behaved normally. The retardation of S1 descendants (Figure3e) resembles that in the manipulated *A. nanus* embryos; however, a separation into AB and EMS fates (indicated by muscle contractions and gastrulation events) was not found. As our observations indicate an influence of P1 or its descendants on the behavior of S1 cells, we investigated to what extent specification of pharyngeal cells depends on inductions as in *C. elegans*.

Variable cell contacts indicate differences in specification of pharyngeal cells. In the monomorphic *C. elegans*, the pharynx is generated in a complex polyclonal way by a fixed subset of S1 (ABara and alp) and S2a (MS) descendants. Pharynx precursor cells enter the body cavity from the ventral side during late gastrulation forming a cylinder anterior of the intestine [14]. Specification of S1-derived cells that contribute to the pharynx requires signaling from S2a descendants [25]. We analyzed the situation in *P. sambesii* in order to explore to what extent cell specification in the detected polymorphs may differ from *C. elegans*.

Our cell lineage analysis revealed that the pharynx in *P. sambesii* is composed polyclonally of S1 (AB) and S2a (MS) descendants comparable to *C. elegans*. Only in the former, the extending blastopore forms a prominent furrow (Figure1h-j) through which pharynx precursors (descendants of S2a) are translocated into the center of the embryo. Cells on the margin of this furrow contributing to the pharynx belong to four of the eight S1 clones. However, their lineage origin varies in the R, M1, M2 and L polymorphs described above (Figure4, bottom). Hence, we conclude that in *P. sambesii,* the origin of the S1 contribution to the pharynx is not related to cell lineage origin and therefore must depend on cell position.

**Figure 5:**
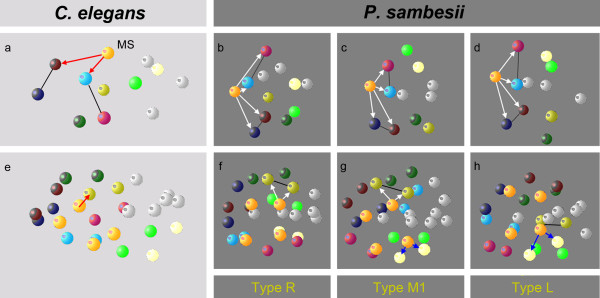
**Cell contacts of S2a cells.** Three-dimensional models of embryonic cell nuclei. In the 12-cell stage in *C. elegans*, S2a (MS) contacts and induces two of the four S1a descendants (**(a)**; red arrows). In *P. sambesii* in the three different spatial variants, S2a (MS; orange) contacts all four S1a (ABa) descendants (**(b-d)**; white arrows). In the 26-cell stage in *C. elegans,* MSap contacts and induces a single AB descendant (**(e)**; red arrow). In *P. sambesii* either both (**(f, g)**; white arrows) or none **(h)** of S1 (AB) sister cells contacts the corresponding S2a (MSa) cell. Black bars connect selected sister cells. For nomenclature of spatial variants, see Figure 4.

The corollary that major differences must exist between *C. elegans* and *P. sambesii* with respect to cell specification is supported by the pattern of cell-cell contacts in the early embryo of the former. In the 12- and 26-cell stages, respectively, two inductions of single AB descendants by MS (Figure5a, e) lead to different fates of originally equipotent cells [25]. In contrast, in *P. sambesii*, S2a (MS) contacts in the 12-cell stage all four descendants of S1a (ABa) in the polymorphs R, M1 and L (Figure5b-d) and in three of these in the variant M2 (not shown). In the 26-cell stage either both sisters (Figure5f, g) or none (Figure5h) is touched by the corresponding S2a (MS) cell. Hence, cell contacts in *Plectus* are not compatible with a mechanism of cell specification as found in *C. elegans*.

With the observed embryonic polymorphs in mind (Figures 4, 5) leading to differences in pharynx formation, we investigated how hypodermis is generated in *P. sambesii*.

### Specification of S1-derived hypodermis depends on cell position

In *C. elegans* the hypodermis of the hatching juvenile is generated in a reproducible manner by members of the S1 (AB) and S3 (C) lineages [14].

**Figure 6:**
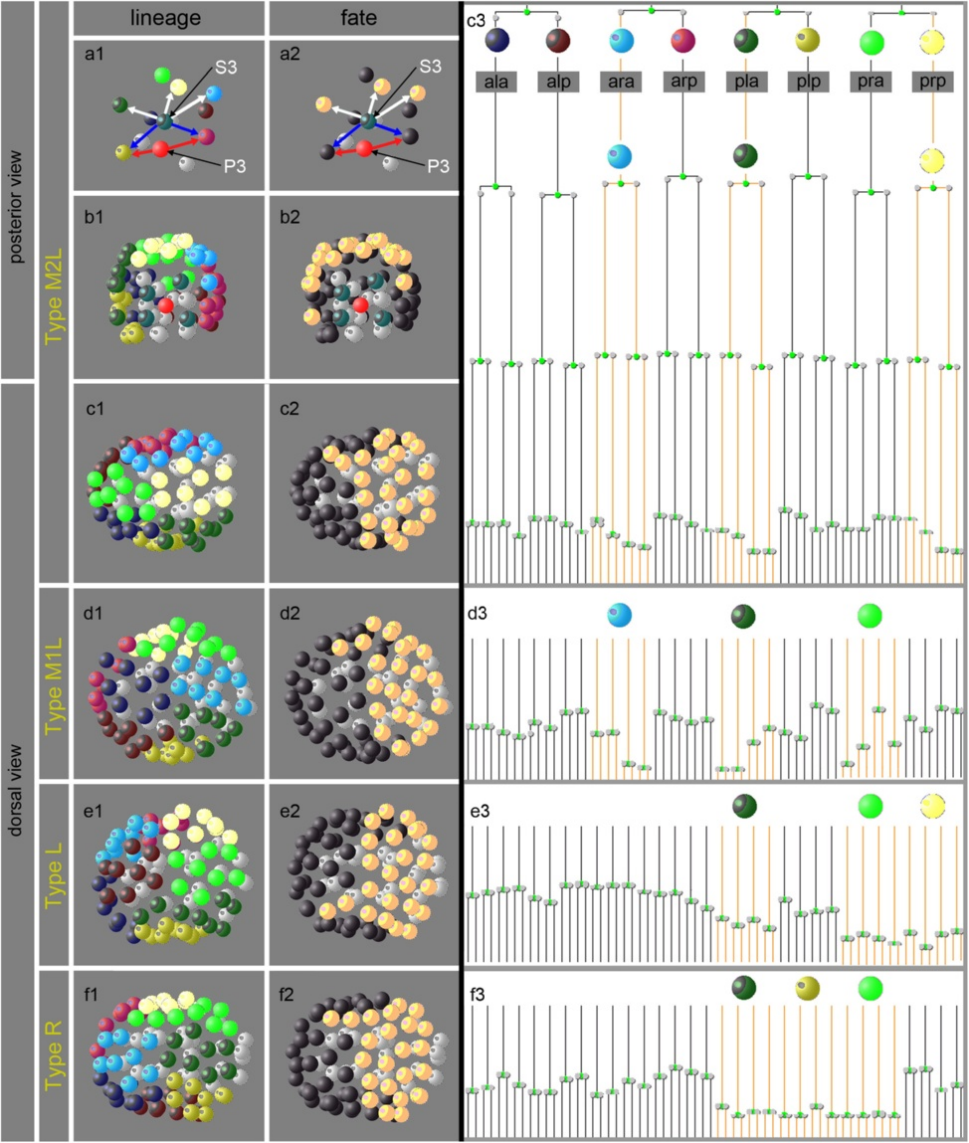
**Different lineage, same fate. (a1-f2)** Three-dimensional models of embryonic cell nuclei. In early *Plectus* embryos, five S1 descendants are contacted by S3 (white and blue arrows). Two of these are contacted in addition by P3 (red arrows). The three S1 blastomeres contacted exclusively by S3 (white arrows) generate descendants that occupy posterior dorsal and posterior lateral positions (b1, c1). These cells (light orange; remainder of S1, black) contribute to hypodermis (a2-c2) and later express retarded cell cycles (c3). In cleavage types M1L, R and L, different sets of three S1 cells are exclusively contacted by S3 (not shown). Nevertheless, they all occupy the same positions as those in type M2L (d1-f1), contribute to posterior hypodermis (d2-f2) and express retarded cell cycles (d3-f3). For nomenclature of spatial variants, see Figure 4.

Lineage analysis (n = 13) revealed that in *P. sambesii*, like in *C. elegans*, hypodermis is derived in a polyclonal manner from S1 (AB) and S3 (C) descendants. We found that in the embryonic polymorphs R, M1, M2 and L, descendants of S1 contributing to hypodermis always occupy the same spatial position (Figure6b2-f2), which means that they have different lineage origin (Figure6b1-f1). Only these blastomeres execute a retarded cell cycle rhythm compared to the other S1 descendants (Figure6c3-f3). During ongoing development, they remain on the surface and some (positioned left and right of the dorsal midline) eventually interdigitate (not shown), typical for hypodermis formation in *C. elegans*[14] and *R. culicivorax*[16,32].

We revealed that in all observed polymorphs, only the S1 descendants in contact with S3 but not with P3 expressed a slower cell cycle (Figure6a1, c3-f3). To test whether this was due to an induction, we performed a series of cell ablation experiments.

After irradiation of S3 (n = 2) or their ancestors P1 (n = 3) and P2 (n = 3), the typical retardation of posterior S1 descendants did not take place (Figure3q) and no cells with hypodermis-like characteristics (see Methods) formed. In contrast, normal formation of hypodermis took place after ablation of S2a (data not shown). Hence, our results indicate that specification of hypodermis from variable posterior S1 cells takes place in a position-dependent manner and involves signaling by S3.

### Variable chirality during early cleavage but invariant handedness of adults indicates position-dependent cell specification

**Figure 7:**
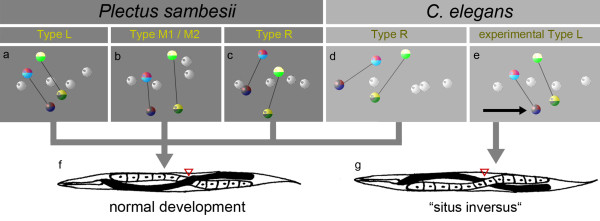
**Position of early S1 (AB) descendants and handedness of adults. (a-e)** Three-dimensional models of embryonic cell nuclei. In *C. elegans*, the four descendants of S1 form an early left/right asymmetry, (d) resulting in a specific arrangement of gut and gonad (**(f)**, ‘normal development’). Experimentally induced mirror-image arrangement of S1 descendants (e) results in a ‘situs inversus’ **(g)**. In *Plectus sambesii*, positions of S1 cells differ considerably (a-c), but all variants develop into adults with normal handedness (f). For nomenclature of spatial variants, see Figure 4.

Early cell arrangement in the prevailing polymorph R (Figure4, left column; Figure7c) resembles the invariant pattern found in *C. elegans* (Figure7d; [26]).In previous experiments, Wood [27] generated an artificial type L via micromanipulation of early AB blastomeres (Figure7e). In contrast to normal development (Figure7f), this variant gave rise to a ‘situs inversus’ with mirror-image orientation of organs (Figure7g). To test whether any of the various polymorphs of *P. sambesii* developed abnormal handedness, we analyzed a large number of adults (n >200) for the presence of situs inversus.

All specimens showed the standard left-right asymmetries (Figure7f) and not a single situs inversus was detected. This allows the conclusion that in the *P. sambesii* embryo, all of the identified polymorphic variants merge into a single adult phenotype. This finding gives further support to the conclusion drawn from the other observations reported above, that is, that in contrast to *C. elegans*, cell specification of S1 descendants depends on position in the embryo rather than position in the lineage tree.

### A shift from polymorphic to monomorphic embryogenesis supports a subdivision of the taxon Chromadorea

**Figure 8:**
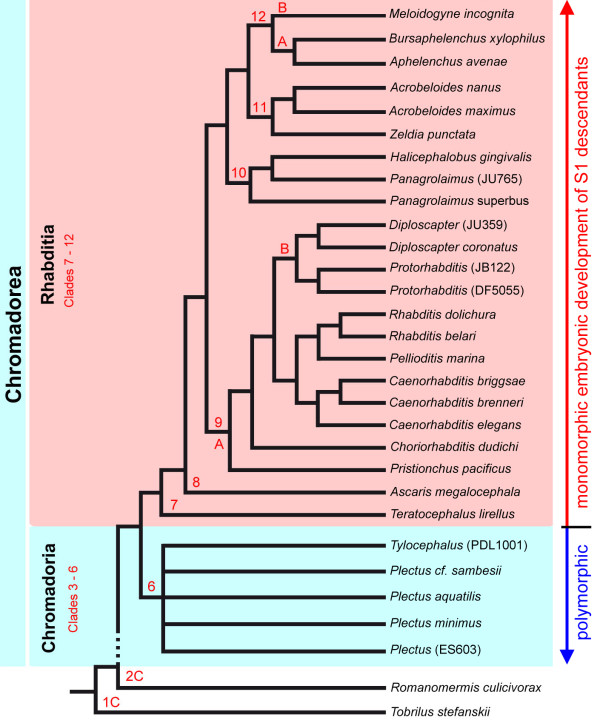
**Phylogeny and development.** Phylogenetic tree of nematodes ([19], modified [17]) comprising 12 clades. In the 23 studied members of clades 7 to 12, an invariant monomorphic development of S1 descendants was found, while all five representatives of clade 6 follow a variable polymorphic pattern. Based on these findings and additional data referenced in the text, a separation of ‘Chromadorea’ into ‘Rhabditia’ and ‘Chromadoria’ is supported.

As we had found early polymorphs in *P. sambesii* embryos associated with major differences in cell specification compared to the monomorphic *C. elegans*, we wondered how other members of clade 6 would behave. Analysis of three additional *Plectus* species and one *Tylocephalus* (each n ≥7; Figure8) made clear that they all form variable spatial patterns of S1 descendants very much like those found in *P. sambesii* (data not shown).

Studies in other representatives of clades 8 and 9, for instance *D. coronatus*[49], *P. marina*[29] or *C. briggsae*[50], revealed that these are monomorphic and that development is very similar to that of *C. elegans*.

**Figure 9:**
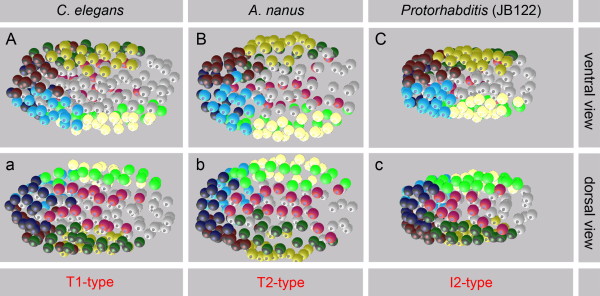
**Cleavage type and cell pattern.** Three-dimensional models of embryonic cell nuclei. A very similar arrangement of 64 S1 descendants is found in three nematode species with different early cleavage patterns (T1, T2, I2; see [17]). A-C, ventral view; a-c, dorsal view. Orientation: anterior, left. For color code, see Figure 1.

Since we had previously found four different early embryonic cleavage variants (T1, T2, I2 and I3; [17]) among species of clades 7 to 12, we explored whether these show polymorphic behavior as described above. For this, we studied 22 additional members of these clades (each n ≥7; Figure8) and found that embryos, despite distinct spatial variations during initial cleavage stages, all merge into the monomorphic *C. elegans*-like arrangement. Three examples, each representing a different early cleavage pattern [17], are shown in Figure9.

The results from this comparative study support a subdivision of the taxon Chromadorea [21] into species with a *Caenorhabditis*-like development (‘Rhabditia’) and those following a *Plectus*-like development (‘Chromadoria’). Such a separation had been suggested earlier based on morphological and developmental peculiarities in clades 3 to 6 [7,22,51].

## Discussion

### Nematodes of clade 6 appear to follow a different developmental strategy

The study of embryogenesis in 28 nematode species from clades 6 to 12 (according to the phylogeny of [19]) representing different early cleavage patterns demonstrates a high diversity during early development and indicates major evolutionary modifications in the early cleavage and differentiation program of nematodes [17]. Surprisingly, embryogenesis in all 23 species of clades 7 to 12 merges into a common monomorphic (invariant) cell pattern prior to the onset of gastrulation comparable to that described for *Ascaris*[12,13,52] and *C. elegans*[14,31]. The conserved spatial pattern following the initial variant phase can be best explained with the necessity for specific cell neighborhoods to allow inductions essential for proper specification of blastomeres [25]. Initially, founder cells need to be positioned in a specific order along the primary body axis [14,17], then orientation of subsequent cleavages, steric constraints and secondary early cell rearrangements [49] result in a common monomorphic pattern.

However, our findings in *Plectus sambesii* and other representatives of clade 6 with their variable cell localization of S1 descendants appear to reflect a different developmental strategy. These species pass through a polymorphic development with a limited number of spatial variants, which nevertheless result in a single adult phenotype. This excludes a lineage-dependent early cell-specification as found in *C. elegans*[14,25] and gives no indication for a global cell sorting as postulated by Schnabel *et al*. [45].

Early developmental similarities between plectids and selected species of clades 3 to 5 give some support for the assumption that these may share the same cell specification mechanisms [7,53].

### Embryonic cell patterns and establishment of left/right asymmetry

In many bilaterian animals, a left/right asymmetry of their internal organs has been found whereby one variant of asymmetry is predominant while the other (‘situs inversus’) is rare or even absent [54,55]. In a variety of nematode species, a preferred handedness was reported by zur Strassen [56] and explained with Mendelian inheritance. In *Ascaris* as well as in *C. elegans*, this asymmetry is established already in the 6-cell stage embryo when a shift of the left pair of AB cell cousins relative to their counterpart on the right side takes place [12,26,56]. In all analyzed species of clades 7 to 12 (Figure8) we observed only right-handed embryonic asymmetry resulting in a single corresponding adult phenotype. As an experimentally induced inverted embryonic asymmetry in *C. elegans* results in healthy, fertile ‘situs-inversus’ adults [27,28], the dominance of one variant is obviously not due to a developmental necessity but may have been fixed in the population because of steric or genetic constraints [54] or later benefits, like increased mating success as suggested by zur Strassen [56]. So far, only one nematode species has been described where a sinistral embryonic and adult phenotype is standard [57] but studies in other systems, such as snails and mice, indicate that a switch in handedness can be induced by a single mutation [58,59]. In contrast to the nematodes mentioned above, in *Plectus sambesii* we not only identified left and right but also rare intermediate embryonic patterns, which, however, all lead to the same dextral adult phenotype (Figures 4,7). The unusual variation in the arrangement of coherent clonal regions exhibited by plectids with their implications on cell specification invokes the question of what changes must have taken place during evolution to come from a polymorphic, *Plectus*-like to a monomorphic, *C. elegans*-like mode of development and what the driving forces for this may have been.

### Evolution of cell lineage and cell specification - a scenario

In a recent publication [17], we sketched the gradual evolution from basal nematodes of the taxon Enoplida (clade 1) with no detectable early cell lineage [7] and delayed fixation of cell fate [60] via representatives with a partial early lineage as found in *Tobrilus* to *Romanomermis* (clade 2) with invariable development but predominantly monoclonal lineages. For a better understanding of how evolution proceeded to species with a complex polyclonal lineage as found in *C. elegans*[14] and all other studied representatives of clades 7 to 12 [29,50,61] our findings in *Plectus* may be helpful.

Taking for granted that each taxon exhibits a combination of plesiomorphic and apomorphic characters, *Plectus* can be viewed as a stepping stone between basal nematodes (clades 1 to 2) where early specification is absent [60] and more derived representatives (clades 7 to 12; see Figure8) with their invariant, polyclonal cell lineage. While in *Plectus* the descendants of P1 behave invariantly, this is markedly different in the progeny of S1 (AB). However, the variants in this lineage do not show a random distribution. The dextral-type RL is by far the most abundant (Figure4) and this is just the one and only variant present in *C. elegans,* suggesting that, on an evolutionary time scale, *Plectus* may be on its way toward an invariant cleavage pattern as found in *C. elegans*.

We envision four important steps for such a transition to happen. (i) Starting from a nematode with a polymorphic monoclonal cell lineage (so far hypothetical), the introduction of position-dependent inductions initiates the beginning of a polyclonal lineage whose complexity will increase over time. (ii) The initially random distribution of polymorphic variants becomes biased, possibly by steric constraints like changes in shape or size of the eggshell or modified orientation of cleavages. The observed predominance of dextral variants in *Plectus* (Figure4) may serve as an example for this. Compared to *Plectus* (Figure1; [22]), the C. *elegans* embryo is more jammed inside the egg envelope, giving a possible explanation for an increase of situs inversus there after removal of the eggshell [62]. (iii) The essentially perfect bilateral symmetry as found in *Romanomermis*[16,32] and *Plectus* (Figures 2,6; [22]) is broken and compensated by inductions [23-25] resulting in cells that perform equivalent fates in the left and right half of the embryo, although they occupy nonequivalent positions in the lineage tree. A variety of such examples can be found in the AB lineage of *C. elegans*[14]. (iv) The genetic fixation of cell fate via signaling of neighboring cells reduces tolerable variability and eventually leads to a monomorphic pattern. This goes along with an increasingly earlier specification of blastomeres. Elimination of gene function (see www.wormbase.org) and cell isolation experiments [63,64] demonstrate the presence of a tightly woven network of early cell-cell interactions in *C. elegans*.

#### *Lineage versus position. How big is the difference between Plectus and C. elegans?*

The highly variable patterns of S1 cells in *Plectus* demonstrate the requirement of cell-cell interactions for proper cell specification. How fundamental then is the difference to *C. elegans*? The classic view of a purely cell-autonomous intrinsic cell specification in nematodes [42,65] has been overcome with the discovery of early cell-cell interactions in *C. elegans* (for recent review, see [66]) which include S1 cells. A strong argument that in *C. elegans* fate assignment in the S1 lineage must also depend on extrinsic inductions beyond the cases revealed so far is supplied by the finding that an isolated S1 cell generates only surviving descendants that contribute to the nervous system when cell-cell signaling is suppressed [45,67]. Promising candidates for further inductions in the S1 lineage include, for instance, anterior descendants with equivalent positions in the left and right half of the embryo but nonequivalent lineage affiliation, which nevertheless execute equivalent, bilateral symmetric fates [14]. Thus, the prominent difference between both species with respect to cell specification of S1 cells can be considered a gradual and not a fundamental one. The variable pattern formation going along with late, position-dependent fate assignment as found in *Plectus* appears to be a plesiomorphic character predominant in certain basal nematodes but lost in representatives of clades 7 to 12. In this respect, *Plectus* and its kin may be in a transition phase between two different developmental strategies.

### Evolution of cell lineages and gene regulatory networks

The driving forces for the establishment of a *C. elegans*-like cell lineage mechanism could be a faster developmental tempo due to an increased recourse to maternal gene products, an improved cost-efficiency of embryogenesis [68] or a better reliability of the cell-specification program, resulting in higher reproductive success. It has been argued that a monoclonal lineage allows a simple way for cell specification but requires extensive cell migrations, whereas a polyclonal lineage means more cell specification decisions but allows cells to be born where they are needed [29,69]. Both strategies have their advantages, but when rapid development and low cell numbers are an issue, a polyclonal invariant development should be superior. In addition, subdivision of a lineage into many small modules offers increased options for evolutionary fine-tuning. Thus, a polyclonal lineage offers more flexibility in the course of phylogeny, but less during ontogeny [32].

Studies on embryonic cell specification [10] vulva formation [11,70] and sex determination [71] in nematodes of the same clade as *C. elegans* revealed major modifications with respect to the underlying gene regulatory networks. Changes in the subcellular control mechanisms of development without corresponding reflections on the anatomical level, called developmental system drift [72], appear to be a common process in the animal kingdom, indicating that selective pressure is low on how to make an organism, but high on the functional capability of the final product. The cellular variations we report here together with previous studies [7,17,32,73] suggest that even more significant alterations of the molecular machinery can be expected when representatives of the whole phylum are compared. Initial support for this comes from genomic and gene expression data of various nematode species ([74,75]; our unpublished results). Nematode embryogenesis taking place in the protected encasement of a rigid egg envelope appears to be a fertile field for the exploration of alternative developmental pathways.

## Conclusions

Our embryonic studies in nematodes of the genus *Plectus* reveal major differences with *C. elegans*. Most prominent is the variable positioning of S1 (AB) descendants, implying a position- rather than lineage-dependent mode of cell specification. With this peculiarity, *Plectus* appears to occupy an intermediate position between basal nematodes following an indeterminate early development and the *C. elegans*-like invariant pattern. Our results exemplify that developmental system drift not only allows large-scale modifications of gene regulatory pathways without impact on morphology but also of blastomere behavior during embryogenesis. While nematodes seem to be particularly suitable for studying the evolution of development due to their long history, ubiquitous distribution and amenability for detailed lineage analysis, the challenge for the future will be to correlate the alteration of cell behavior with the dynamics of the underlying molecular scaffolding.

## Abbreviations

DSD, developmental system drift; EGM, embryonic growth medium.

## Competing interests

The authors declare that they have no competing interests.

## Authors’ contributions

JS, WH and ES contributed to the conception and design of the study, all authors were involved in acquisition of data, its analysis and interpretation. JS and ES drafted the manuscript and all authors read and approved the final version.
